# Description of adult and third instar larva of *Trichostetha curlei* sp. n. (Coleoptera, Scarabaeidae, Cetoniinae) from the Cape region of South Africa

**DOI:** 10.3897/zookeys.428.7855

**Published:** 2014-07-23

**Authors:** Renzo Perissinotto, Petr Šípek, Jonathan B. Ball

**Affiliations:** 1 Department of Zoology, Nelson Mandela Metropolitan University, PO Box 77000, Port Elizabeth 6031, South Africa; 2 Department of Zoology, Charles University in Prague, Viničná 7, CZ- 128 44 Praha 2, Czech Republic; 3 Department of Zoology & Entomology, University of Pretoria, Pretoria 0002, South Africa

**Keywords:** Scarabaeidae, Cetoniinae, genus *Trichostetha*, new species, revised status, third instar larva, Cape Floral Region, South Africa

## Abstract

A new high altitude montane species of *Trichostetha* Burmeister, 1842 is described from the Elandsberg range of the Western Cape interior. This represents the 14^th^ species of the genus and the first to be reported with a description of its larva. It is a significant addition to the growing number of species that exhibit no adult feeding behaviour and a short period of activity restricted to the onset of summer. Larvae dwell in rock crevices, feeding on decomposing plant matter. The genus *Trichostetha* is heterogeneous and the complex variability observed in some species, especially *T. capensis* (Linnaeus, 1767), requires the re-instatement of taxa that were recently synonymised. Thus, *T. bicolor* Péringuey, 1907 is here re-proposed as a separate species and *T. capensis hottentotta* (Gory & Percheron, 1833) as a separate subspecies. Conversely, *T. alutacea* Allard, 1994 is recognised as a dark variety of *T. signata* (Fabricius, 1775) and is, consequently, synonymised with this species.

## Introduction

The genus *Trichostetha* Burmeister, 1842 is endemic to southern Africa and currently consists of 13 species and four subspecies (Holm and Marais 1992; Holm and Stobbia 1995; Sakai and Nagai 1998; Holm and Perissinotto 2004). With the exception of *Trichostetha fascicularis* (Linnaeus, 1767), which is subdivided into a number of subspecies and is widespread throughout South Africa and in the southern part of Botswana (Holm and Perissinotto 2011), all the other species are fairly stable and restricted to small distributional ranges (Holm and Marais 1992). Most of them occur in the Cape Floral Region and generally, but not exclusively, in mountainous terrain. Adults have traditionally been reported as feeding on the flowers of a variety of *Protea*, *Berkheya* and *Berzelia* (Péringuey 1907; Holm and Marais 1988; Holm and Stobbia 1995; Holm and Perissinotto 2004) as well as *Leucospermum* and *Brunia* species (JB Ball, pers. observ.). However, it is now clear that some species are actually associated with different plant species (e.g. *Trichostetha coetzeri* Holm & Marais, 1988 and *Trichostetha fascicularis maraisi* Stobbia, 1995), while others do not seem to feed at all in their adult stage (i.e. *Trichostetha dukei* Holm & Marais, 1988, *Trichostetha hawequas* Holm & Perissinotto, 2004, *Trichostetha curlei* sp. n. and *Trichostetha calciventris* Stobbia, 1995).

Several species in this genus have only been described recently, from poorly sampled areas or from isolated mountain peaks (Holm and Perissinotto 2004). Observations in these areas have escalated during the past few years and as a result another undescribed species has now been discovered in the Cape region. The larval stages of a few species have been collected and successfully reared in the recent past (Holm and Stobbia 1995, R. Perissinotto pers. observ., A.P. Marais pers. comm.), but to date they have not been described for any of the known species. The new species reported here was collected both in the adult and third instar larval stage. This provides an opportunity to describe for the first time the larva of a representative of this genus.

The objective of this work is, therefore, to present a complete description of adult, larva and ecology of this new species and to relate this to the complex ecological and taxonomic issues that characterize the genus *Trichostetha*.

## Materials and methods

The first records of this new species were reported from December 2009 and November 2011 by HC Ficq, who observed a few specimens in flight or perched on leaves and flowers of typical fynbos vegetation on the southern slope of the Elandsberg range of the Western Cape interior. After it became clear that an undescribed species was involved, a dedicated survey was undertaken to the area in November 2013. All specimens were collected in the mid morning to early afternoon hours using a standard net or direct hand picking. Larvae were obtained by excavating 10–20 cm underground in rock crevices, using standard garden tools. Specimens were preserved in 10% formalin and 99% ethanol for laboratory analyses.

The description of morphological characters follows the terminology used by Krikken (1984) and Holm and Marais (1992). Specimen length was measured from the anterior margin of the clypeus to the apex of the pygidium. Specimen width represents the maximum width of the elytra. Photos of male and female types were taken with a Ricoh CX1 camera with macro setting. Where necessary, the background was removed from the photos using Adobe Photoshop, in order to increase clarity of resolution. In situ photos were taken with a Nikon D800, fitted with 105 mm Nikon macro-lens.

The terminology for larval morphology follows Hayes (1929), Böving (1936) and Ritcher (1966). In order to give the most accurate information on chaetotaxy, the hair-like setae of the cranium and other structures were classified according to their relative sizes into two groups, medium to long (80–300 μm) and minute or small setae (5–40 μm). Refer to Šípek et al. (2008) for a detailed schematic figure. Morphological analysis and measurements were carried out using Olympus SZX9 and Olympus BX 40 light microscopes, both equipped with digital camera Olympus Camedia 5060. Mouthparts were dissected and if necessary mounted on slides in Liquide de Swan (e.g. Švácha and Danilevsky 1986). Photographs were taken using a Canon 550D digital camera equipped with Canon MP-E 65/2,8 MACRO lens with 5:1 optical magnification. Final images were composed from multiple layer-focussed pictures using Helicon Focus software (Helicon Soft Ltd.). All pictures were digitally enhanced using Adobe Photoshop CS4 (Adobe Inc.).

Collections are abbreviated as follows: ISAM – Iziko South African Museum, Cape Town, South Africa; PCBM – Private Collection JB Ball & AP Marais, Cape Town, South Africa; PCRP – Private Collection R Perissinotto & L Clennell, Port Elizabeth, South Africa; PCAC – Private Collection AI Curle, Johannesburg, South Africa; TMSA – Ditsong National Museum of Natural History (ex Transvaal Museum), Pretoria, South Africa. Geographical abbreviations are as follows: WC – Western Cape Province; EC – Eastern Cape Province; NC – Northern Cape Province; FS – Free State Province; KZN – KwaZulu-Natal Province; NW – North-West Province; MP – Mpumalanga Province; LP – Limpopo Province; BO – Republic of Botswana.

## Species description

### Trichostetha curlei sp. n.

Taxon classificationAnimaliaColeopteraScarabaeidae

http://zoobank.org/538D4B0C-0B02-41E8-9608-E5228317F7AC

#### Type series.

Holotype ♂: South Africa, WC, Elandsberg 33°19'49"S, 21°18'13"E, 1478 m asl, 13 Nov 2013, JB Ball & R Perissinotto (ISAM). Paratypes, 16♂ 2♀: same data as above (ISAM, PCBM, PCRP); 5♂ 1♀, same locality, but 16 Nov 2009, HC Ficq (PCAC); 2♂ 2♀, same locality, but 14 Nov 2011, HC Ficq (PCAC).

#### Etymology.

The species is named after the South African lepidopterist AI Curle, who brought to our attention the existence of this undescribed Cetoniinae from the Elandsberg range.

#### Description of adult

**Figure 1.**
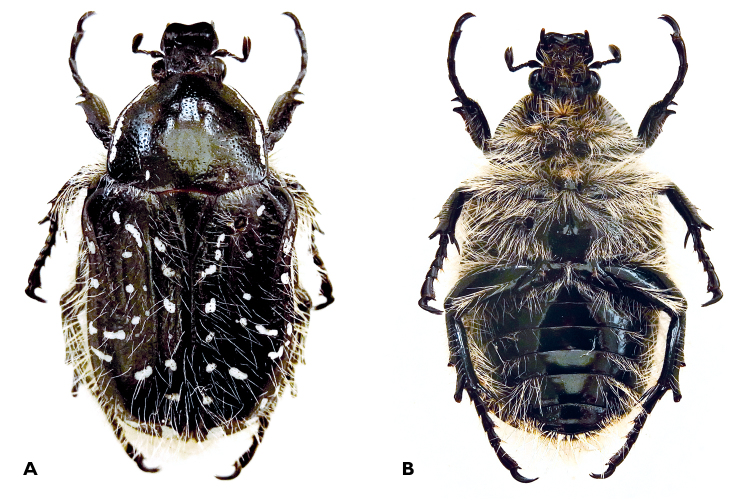
*Trichostetha curlei* sp. n.: Male dorsal (**A**) and ventral (**B**) habitus (length 18 mm) (Photos: L Clennell).

**Figure 2.**
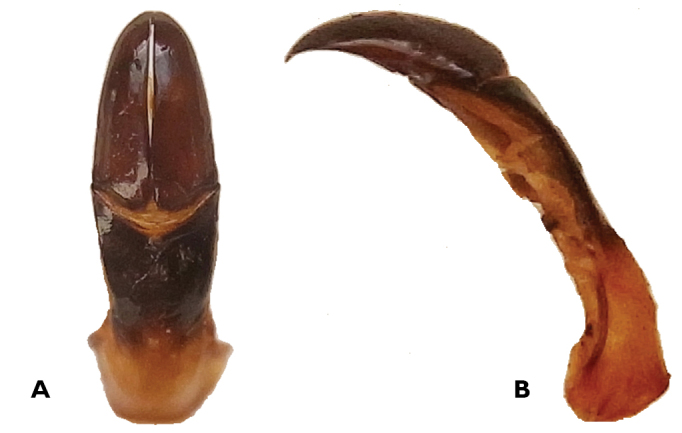
*Trichostetha curlei* sp. n.: Dorsal (**A**) and lateral (**B**) view of aedeagus (length 6.8 mm) (Photos: L Clennell).

**Figure 3.**
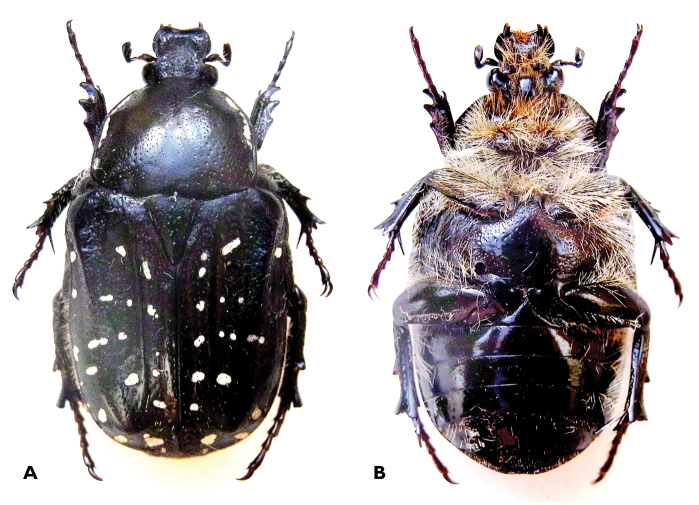
*Trichostetha curlei* sp. n.: Female dorsal (**A**) and ventral (**B**) habitus (length 17.3 mm) (Photo: L Clennell).

**Figure 4.**
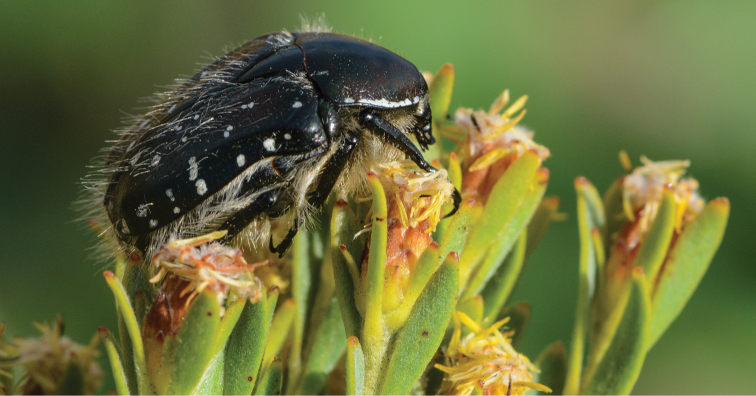
*Trichostetha curlei* sp. n.: Male specimen in its natural habitat on the Elandsberg summit, November 2013 (Photo: JB Ball).

(Figures 1–4). ***Male, holotype.*** Size: length 18.0 mm; width 9.2 mm.

*Body* (Figure 1A). Dark green to black; long white setae and white tomentose marks widespread, particularly on elytral surface.

*Head*. Black with scattered whitish, long setae and dense round punctures; clypeus sharply upturned, with broad anterior margin rounded, depressed at centre, forming two lateral lobes, which together with marked lateral ridges form concavity with raised frons at middle; antennal club, pedicel and flagellum black; antennal club short, pedicel and flagellum with long, erected white setae.

*Pronotum*. Dark green to black, shiny; disc without setae and with only few small, shallow punctures; scattered round punctures becoming larger and denser towards lateral margins; short setae restricted to lateral declivity; white tomentum restricted to stripe on lateral margins and two basal spots near posterior margin in shallow depression anteriad of scutellar basal corners; postero-lateral angle slightly rounded, posterior margin mildly sinuate anteriad of scutellum; anterior margin straight and as wide as eyes.

*Scutellum*. Dark green, shiny with sharply pointed apex and deep lateral grooves; few, scattered small punctures bearing short setae mainly around basolateral corners.

*Elytron*. Dark green to black; with sparse but very long white setae; humeral and third costa sharply raised and very marked; umbone wider than pronotum; small sparse white maculae, denser on second and fourth elytral striae where they form dotted lines, larger maculae present on lateral and apical declivity; crescent to horseshoe punctures distributed throughout surface, becoming more irregular in shape and scattered on umbone, costae, lateral and apical declivities.

*Pygidium*. Black with long white setae; with extensive white markings in basal two thirds, on each side of midline.

*Legs*. Protibia with few short setae, tridentate, with proximal denticle drastically reduced; distal teeth very pointed; anterior arolium bisetose; mesotibia with many long setae and two median external denticles, proximal extremely reduced, distal furcate and pointed outwardly; mesotibial claws sharply bent to form hook-like structure; metatibia covered in long setae, with median outer ridge not very pronounced, posterior arolium bisetose; all femora covered in dense, long white setae.

*Underside* (Figure 1B). Dark green, shiny, with dense setation and scattered punctures covering entire surface, except parts of mesometasternal process, medial half of abdominal sternites and ventral surface of metafemora; mesosternal lobe simple and flat, not protruding; sternites 3-6 with deep medial concavity.

*Aedaegus* (Figure 2). Parameres tapering gradually into sharply pointed apical end, gently curved downwards at apical half; without setae.

***Female, paratype*** (Figure 3). Size: length 17.3 mm; width 8.9 mm. General shape and colour patterns similar to male, but white marks substantially reduced throughout body surface; white setation drastically reduced both on dorsal and ventral surfaces; tarsal segments shorter and protibial denticles wider and rounded; wing surface approximately one fifth smaller than in male; sternites 3-6 with slight medial convexity.

#### Variability.

Males range from 12.9 to 18.0 mm in length and from 7.0 to 9.0 mm in width (n = 24), while females vary between 15.5 and 17.3 mm in length and between 8.2 and 8.5 mm in width (n = 2). All specimens exhibit a dark green to black dorsal colour, but there are brown areas on elytral disc in some cases. The extent of white maculation throughout the surface can also change substantially among specimens. The male pronotum occasionally exhibits second and/or third smaller discal spots in shallow depressions towards the lateral declivity, at middle or near the anterior margin.

#### Diagnosis.

*Trichostetha curlei* sp. n. is most closely related to the smaller species of the genus, *Trichostetha dukei* and *Trichostetha hawequas*. It differs from both in having extremely well developed white pilosity in the male, sharp elytral costae, dark green background colour and extensive white maculation on the pygidium (Figures 1, 3, 4).

#### Remarks.

It appears that, despite being seen on numerous flowers in different plant families, the univoltine adults are unable to feed and are active for a relatively short period of time at the onset of summer, from mid November to mid December. Their observed behavior is unexpected for a member of the genus *Trichostetha*, in that they show very little flying activity, preferring rather to perch on leaves and flowers of a variety of shrub species (Figure 4), which may serve as mating platform. Adults were noted on a number of plants, including *Leucadendron rubrum* (both male and female flower heads), *Protea eximia*, *Passerina truncata*, *Elegia filacea* (both male and female flowers) and *Cullumia bisulca*.

#### Description of third instar larva

**Figure 5.**
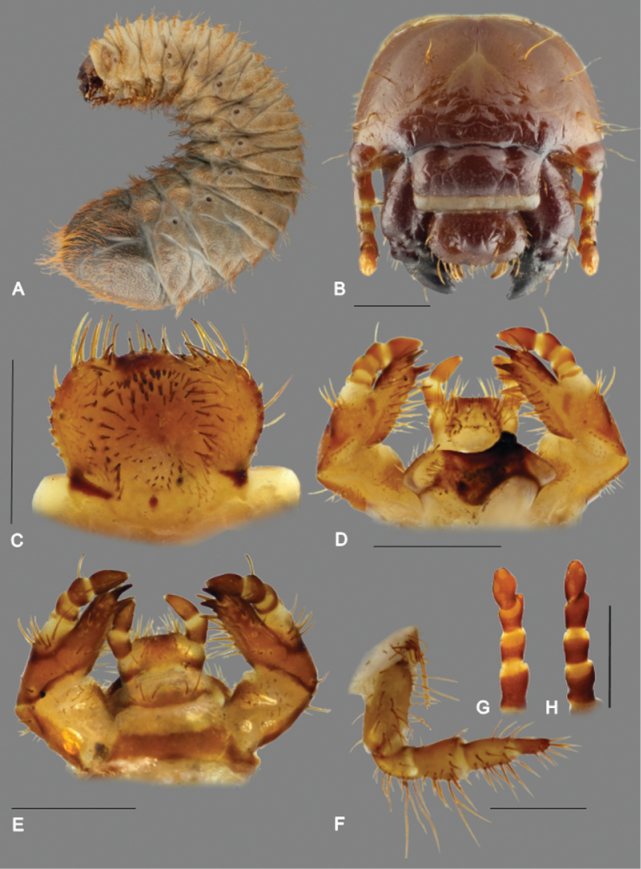
*Trichostetha curlei* sp. n., third instar larva. **A** Habitus of fully grown larva (length 41 mm) **B** cranium **C** epipharynx **D** Labio-maxillar complex and hypopharynx, dorsal aspect **E** labio-maxillar complex and hypopharynx, ventral aspect **F** metathoracic leg, lateral aspect **H** antenna (**G** dorsal aspect **H** ventral aspect). Scale bars: 1 mm (Photos: P Šípek).

**Figure 6.**
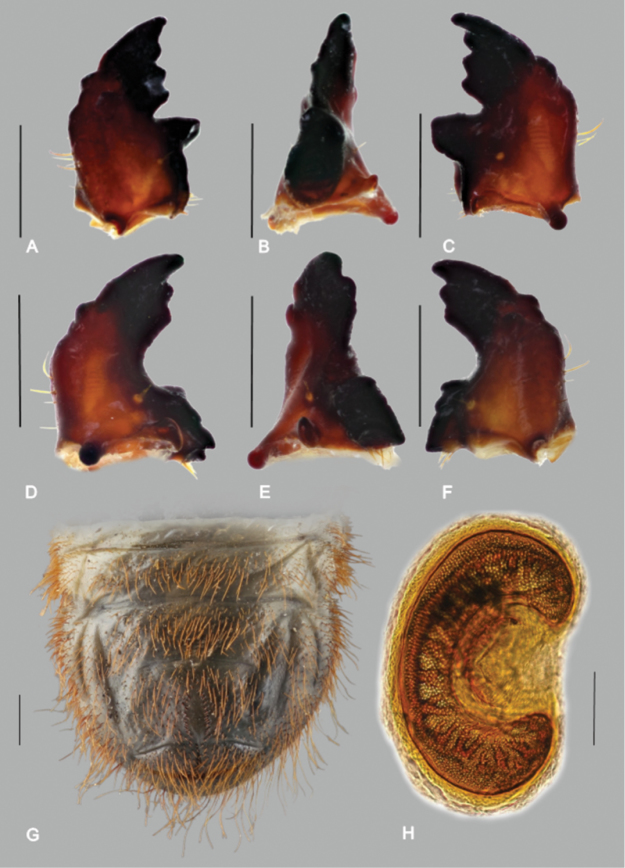
*Trichostetha curlei* sp. n.,third instar larva. **A, B, C** Left mandible (**A** dorsal aspect **B** medial aspect **C** ventral aspect) **D, E, F** right mandible (**D** ventral aspect **E** medial aspect **F** dorsal aspect) **G** last abdominal segments, ventral aspect **H** thoracic spiracle. Scale bars: **A**–**G** – 1 mm, **H** – 0.1 mm (Photos: P Šípek).

(Figures 5–6). ***Material examined.*** Two third instar larvae with the following data: South Africa, Western Cape Province; Elandsberg, 13 Nov 2013, JB Ball & R. Perissinotto legit.

*Body* (Figure 5A). Larva scarabaeiform, of typical Cetoniinae habitus, maximal length 38–41 mm; cranium brown to dark-brown, body densely setose, creamy-white or greyish, setae yellowish to orange-brown; abdominal segments IX and X fused dorsally, ventrally separated by an incomplete groove.

*Head capsule* (Figure 5B). Maximum width between 3.3 and 3.6 mm; cranium glabrous with linear microsculpture, brown; antennifer, postclypeus and labrum darker, frontoclypeal suture and apices of mandibles black; frontal sutures shallowly bisinuated; epicranial insertions of antennal muscles feebly developed (visible only as small depressions near the midline of frontal sutures); epicranial suture extending anteriorly into frons; cranial chaetotaxy summarized in Table 1; posterior epicranial setae with a single long and several minute setae; anterior frontal setae absent, exterior frontal setae minute; clypeus almost rectangular, membranous anteclypeal part covering about 1/3 of entire clypeal area; postclypeus strongly sclerotized with one anterior and a pair of exterior clypeal setae (one shorter than the other); frontoclypeal suture distinct; stemmata rudimental.

**Table 1.** Cranial chaetotaxy of *Trichostetha curlei* sp. n. third instar larva.

	Epicranium				Frons				Clypeus		Labrum				
Group of setae	DES	PES	AES	EES	PFS	EFS	AFS	AAS	ACS	ECS	PLS	PMS	ELS	LLS	SMLL
Long + medium	1+1	1+0	1	1-3+8-12	1	0	0	1	1	1+1	0-1+1-3	1	2+1-2	7-8	8
Minute	4–9	2–4		3–4	0	1	0	0	0	0	0	0	0	0	0

Abbreviations: AAS = setae on anterior frontal angle; ACS = anterior clypeal setae; AES = anterior epicranial setae; AFS = anterior frontal setae; DES = dorso-epicranial setae; ECS = exterior clypeal setae; EES= exterior epicranial setae; EFS = exterior frontal setae; ELS = exterior labral setae; LLS = setae on lateral labral lobe; PES = posterior epicranial setae; PFS = posterior frontal setae; PLS = posterior labral setae; PMS = paramedial labral setae. SMLL = seate on the medial labral lobe. For explanation of length categories of setae see 'Materials and methods'.

*Antennae* (Figures 5G, H). Four-jointed (an I–IV), relative length of antennomeres: an I > an IV > an II > an III; an III with ventral, apical projection exhibiting a single sensory spot; roud (apical) sensorial field of ultimate antennomera shifted slightly laterally; two dorsal and two ventral sensorial spots present.

*Labrum*. Symmetrical, anterior margin trilobed with numerous setae; clithra present; dorsal labral surface with two transverse rows of setae.

*Epipharynx* (Figure 5C). Haptomerum with zygum convex, with a transverse row of 12-15 spiny setae, eight to ten similar setae at posterior base of haptomerum; sensilla of zygum grouped below apex of haptomerum; haptomeral process and proplegmata absent; acroparia with external margin of lateral labral lobes with six long setae on ventral and four setae on dorsal side; medial labral lobe with two rows of four setae; acanthoparia bearing five to seven setae with distinctly swollen base (projecting out of epipharyngeal margin); lateral margin of epipharynx serrate; subapical seta of acanthoparia slightly longer than posterior setae, apical seta about three times longer than subapical seta; plegmata absent; chaetoparia with about 60 setae arranged in three to four irregular rows, medial rows with stout, spine-like setae; dexiotorma robust, straight; right pternotorma present; laeotorma short, left pternotorma triangular, large; haptolachus with sense cone (left nesium) low and obtuse, sclerotised plate (right nesium) absent; plate-shaped sclerite feebly developed; anterior part of haptolachus (distad to sense cone) with short spine-like setae; postero-lateral part with group of two pore-like setae on each side; phoba and crepis absent.

*Mandibles* (Figure 6A–F). Asymmetrical, scrobis with four to six setae; longitudinal furrow absent; anterolateral portion of dorsal mandibular surface with two setae (often broken); interior seta more or less distinct, exterior seta short, indistinct; patches of two to four dorsomolar and ventromolar setae concealed in single rim on both mandibles (however, dorsomolar setae were absent on one of two left mandibles examined); stridulatory area with seven to ten transversal ridges; left mandible with four and right mandible with three prominent scissorial teeth; apical third of external mandibular margin with prominent obtuse or slightly pointed tubercle; molar lobes of both mandibles with sharp projections; posterior margin of right madibular calyx bilobed (in medial aspect), dorsal lobe about twice as large as ventral; calyx of left mandible flattened with convex posterior margin; brustiae with 5–7 and 10–13 setae on right and left mandible, respectively.

*Maxilla* (Figures 5D, E). Dorsal surface of cardo and labacoparia with 10–14 and 12–13 setae, respectively; ventral surface of cardo with 2–3 and 21–28 setae, respectively; stipes with around 21–31 slender hair-like setae dorsally and 2–3 long stout setae at external margin; stridulatory area composed of 6–8 well sclerotised spine-like stridulatory teeth organized in oblique raw; truncate process (blunt tubercle) present; ventral face of stipes with single prominent seta at proximal margin and transverse, prominent group of seven setae at base of maxillary palpi; galea and lacinia entirely fused forming mala, galeo-lacinial suture indistinct, entirely absent on ventral surface; galear portion of mala with single falcate uncus and 9–11 long hair-like setae in longitudinal rows; lacinial part of mala with one large and one small uncus and a minute conical seta at base of larger uncus; dorso-medial side with 16–20 very long hair-like setae; setae proximal to galear and lacinial unci generally shorter and stouter than other setae; ventral surface of mala with two longitudinal rows of 3–5 stout setae and basal transverse group of 3–4 hair-like setae; maxillary palps four–jointed, penultimate joint with two setae.

*Hypopharyngeal scleroma* (Figure 5D). Asymmetrical with strong protruding and pointed truncate process; tufts of tegumentary expansions (= phoba, *sensu* Böving 1936) present on left lateral lobe, but absent on central portion of hypopharyngeal scleroma (at base of truncate process); two additional tegumentary expansions below medio-lateral margin of hypopharyngeal scleroma; lateral lobes only feebly sclerotized, anterior margin of left lobe with 12 setae, anterior margin of right lobe with 6–9 setae.

*Ligula* (Figure 5D). Dorsal surface with group of approximately 15–20 long hair-like setae on each lateral margin; paramedial longitudinal row of 3–4 stout setae and proximal transverse row of 12–14 campaniform sensilla interrupted by central pair of conical setae; labial palpi two–jointed.

**Figure 7.**
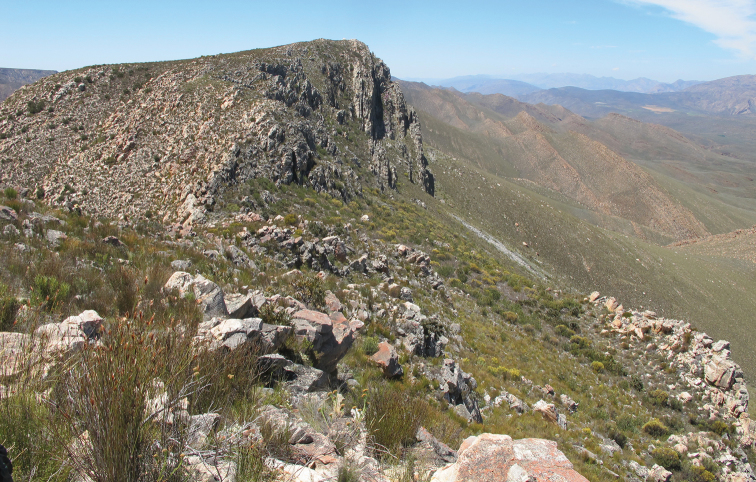
*Trichostetha curlei* sp. n.: Typical habitat on the southern slope of the Elandsberg range (Photo: R Perissinotto).

*Thorax* (Figures 5A, F; 6H). First thoracic sublobe densely setose, with approximately 12 transverse rows of setae; posterior margin of prothoracic sclerite with about 10 setae on dorsal and four setae on ventral surface; each sublobe of meso- and metathoracic segments with 3–6 rows of dorsal setae, anterior row(s) generally with shorter setae than posterior rows; respiratory plate of mesothoracic spiracle “C”-shaped, 0.4 × 0.25 mm (height × width) (Figure 7H); distance between lobes almost equal to maximal diameter of respiratory plate; respiratory plate with 15–20 holes across diameter; all pairs of legs subequal (Figure 5A, F); pretarsi cylindrical with 7–8 setae and minute pointed tip.

*Abdomen* (Figures 5A, 6G). Nine-segmented, densely setose; dorsi of abdominal segments I–VI with three sublobes, VII and VIII with only two; each sublobe bearing six to ten rows of setae; like in thorax, setae in anterior rows shorter than in posterior rows; abdominal spiracles more concealed than mesothoracic spiracle, distance between lobes of respiratory plate equal or shorter than half maximal diameter of respiratory plate; all spiracles subequal in size; abdominal spiracles on segment VI–VIII more circular than preceding ones; dorsum of last abdominal segment entirely covered with medium to long hair-like setae; hammate setae absent.

*Raster* (Figure 6G). Palidium monostichous (however few irregular pali scattered around main row and on ventral anal lobe), composed of two rows of approximately 15-19 short, spine-like pali; septula posteriorly open, about three times longer than wide; tegilla not separated from other groups of setae, entire venter of last abdominal segment covered with hair-like setae interspersed with few spine-like setae; ventral and dorsal anal lobes with numerous medium to long setae, transverse anal row of dense setae absent.

## Discussion

*Trichostetha curlei* sp. n. represents the latest discovery within a group of montane relict species that have been described recently from the Cape region of South Africa. The high altitude habitat of the Elandsberg range (about 1500 m asl), where the species occurs, is characterized by quartzite fynbos, which comprises about 10% of the Fynbos Biome and is confined to the more arid areas of the Capensis Region (Figure 7). The vegetation unit is specifically Matjiesfontein Quarzite Fynbos (type FFq 3 of Mucina and Rutherford 2006) and includes mainly medium dense and tall shrubs with asteraceous, ericaceous, proteoid and restioid fynbos plants, but also some elements of the Succulent Karoo Biome (Figure 7) (Mucina and Rutherford 2006).

The species most closely related to *Trichostetha curlei* sp. n. are *Trichostetha dukei* and *Trichostetha hawequas*. *Trichostetha curlei* sp. n. differs from the first mainly by its sharply upturned anterior clypeal margin and from the second by its dark green background body colouration and the presence of extensive white markings on the pygidium. The two species also occupy habitats that are remarkably different to that of *Trichostetha curlei*, with *Trichostetha dukei* restricted to South Hex Sandstone Fynbos (FFs 8 of Mucina and Rutherford 2006) and *Trichostetha hawequas* found in Hawequas Sandstone Fynbos (FFs 10 of Mucina and Rutherford 2006) vegetation types. The three are, however, sufficiently different in terms of external morphology (e.g. small body size, variable extent of female brachyptery) and habitat/ecology (e.g. non-feeding adult) from the other *Trichostetha* species to possibly warrant inclusion into a distinct species group. A full revision is required in this regard, including molecular genetic analyses.

**Figure 8.**
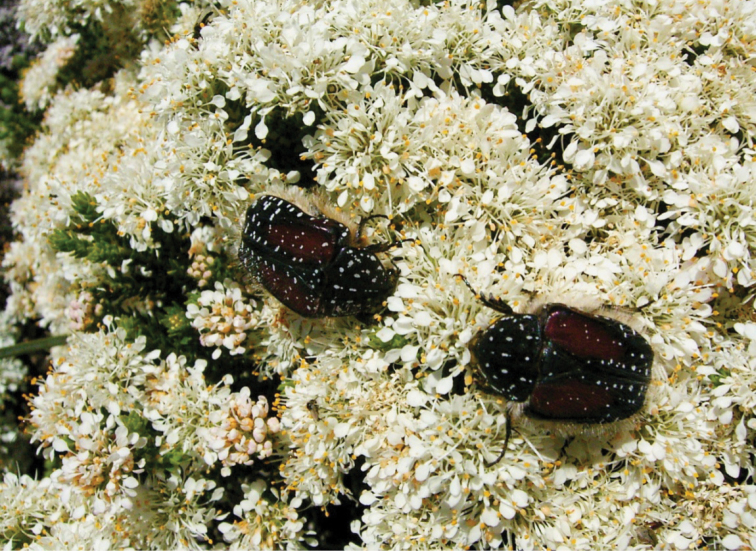
*Trichostetha bicolor* feeding on flowers of *Agathosma capensis* (Rutaceae) at Saldanha Bay, September 2004 (Photo: L Clennell).

The genus *Trichostetha* is complex and includes a number of species and subspecies with as yet unresolved taxonomic status. The largest uncertainties regard the phylogenetic relationships and taxonomic position of the members currently constituting the *Trichostetha capensis* group. Holm and Marais (1988, 1992) synonymized a number of species and subspecies previously described in this group as junior or invalid names of *Trichostetha capensis*. Subsequent observations have shown however that at least two species and two subspecies warrant re-instatement. Antoine (2006) has already argued convincingly for the recognition of *Trichostetha fuscorubra* (Voët, 1779) as a separate species. A review of existing collections and new material gathered during the last 20 years has shown that *Trichostetha bicolor* Péringuey, 1907 should also be regarded as separate from *Trichostetha capensis*. The two species not only differ significantly in phenotypic characteristics, as described by Péringuey (1907), but also occupy different habitats and exhibit different ecological dynamics. Contrary to what was suggested by Holm and Marais (1988), the two neither occur sympatrically nor exhibit intermediate forms. *Trichostetha capensis capensis* is found in the Cape Peninsula area, and possibly further inland to the north, in typical Peninsula Sandstone Fynbos vegetation (type FFs 9 of Mucina and Rutherford 2006), where adults feed mainly on flowers of *Protea*, *Berzelia* and *Leucospermum* species. On the other hand, *Trichostetha bicolor* is restricted to a narrow coastal band between Saldanha and St Helena Bay, where vegetation is very short, of the Saldanha Granite Strandveld type (FS 2 of Mucina and Rutherford 2006). Here, adults feed predominantly on flowers of *Agathosma capensis* (Figure 8).

At the extreme north of its distribution range, in the Cederberg, Piketberg and the Bokkeveldberge, *Trichostetha capensis capensis* is replaced by a distinct subspecies, *Trichostetha capensis oweni*, as described by Allard (1992). The populations located to the east of the Cape Peninsula extend as far as the Kouga mountains of the Eastern Cape and exhibit characteristics broadly matching the description of *Trichostetha hottentotta* of Gory and Percheron (1833), with drastically reduced white maculation and lighter brick-red ground colouration. There are, however, complex variations over this typical form that require molecular analysis, in order to resolve the full taxonomy and phylogeny of the group. It is thus proposed here that a provisional solution may consist of recognizing the subspecies status of *Trichostetha capensis hottentotta*, as suggested earlier by Allard (1992).

*Trichostetha signata* also exhibits some variability, with its southern populations often regarded as a separate subspecies or even distinct species. Recent work undertaken throughout its distribution range has now showed that indeed the population situated in the extreme south-western Cape, especially the Cape Peninsula, represents a valid subspecies with the characteristics originally described by Burmeister (1842) and here re-designated as *Trichostetha signata tibialis*. On the other hand, there is virtual certainty now that *Trichostetha alutacea*, earlier described by Allard (1992) as a proper species, separate from *Trichostetha signata*, is actually a darker, “oiled” variety of the latter. Extensive investigations within the type locality undertaken during the last decade have only revealed the existence of *Trichostetha capensis hottentotta*, *Trichostetha fascicularis fascicularis* and *Trichostetha signata* in the broader area of the Franschhoek Pass. Specimens of *Trichostetha signata* are often darkened by diffusion of lipids to the surface after death, particularly visible when fresh adults are pinned.

It is most likely that new undescribed species occur in the least explored mountain ranges, particularly of the Western Cape, as this seems to be the epicenter of the genus, from where radiation presumably occurred in the past. For instance, the specimen represented in Fig. 1175 (Plate 105) of Sakai and Nagai (1998) is certainly not *Trichostetha calciventris*, as erroneously reported in this book. The locality mentioned for the specimen is “Keeromberg”, which is situated in the extreme western portion of the Langeberg mountain range of the WC. Although a recent search has failed to retrieve further specimens for analysis, it is possible that this may represent a species yet unknown to science, although very closely related to *Trichostetha dukei*.

### Updated list and distribution of *Trichostetha* species and subspecies

*Trichostetha albopicta* Gory & Percheron, 1833; WC, EC

*Trichostetha barbertonensis* Holm & Marais, 1988; MP

*Trichostetha bicolor* Péringuey, 1907; WC

*Trichostetha calciventris* Stobbia, 1995; WC

*Trichostetha capensis capensis* (Linnaeus, 1767); WC, NC, EC

*Trichostetha capensis hottentotta* (Gory & Percheron, 1833); WC, EC

*Trichostetha capensis oweni* Allard, 1992; WC, NC

*Trichostetha coetzeri* Holm & Marais, 1988; NC

*Trichostetha curlei* Perissinotto, Šípek & Ball, sp. n.; WC

*Trichostetha dukei* Holm & Marais, 1988; WC

*Trichostetha fascicularis fascicularis* (Linnaeus, 1767); WC, NC, EC

*Trichostetha fascicularis maraisi* Stobbia, 1995; NC

*Trichostetha fascicularis natalis* Burmeister, 1842; EC

*Trichostetha fascicularis prunipennis* Burmeister, 1842; EC, FS, KZN, MP, LP, NW, BO

*Trichostetha fuscorubra* (Voët, 1779); WC, EC

*Trichostetha hawequas* Holm & Perissinotto, 2004; WC

*Trichostetha mimetica* Devecis, 1997; WC

*Trichostetha potbergensis* Holm & Perissinotto, 2004; WC

*Trichostetha signata signata* (Fabricius, 1775) (= *Trichostetha alutacea* Allard, 1991); WC, EC, NC

*Trichostetha signata tibialis* Burmeister, 1842; WC

## Supplementary Material

XML Treatment for Trichostetha curlei
